# Are Plantarflexor Muscle Impairments Present Among Individuals with Achilles Tendinopathy and Do They Change with Exercise? A Systematic Review with Meta-analysis

**DOI:** 10.1186/s40798-021-00308-8

**Published:** 2021-03-10

**Authors:** Fatmah Hasani, Patrick Vallance, Terry Haines, Shannon E. Munteanu, Peter Malliaras

**Affiliations:** 1 Physiotherapy Department, School of Primary and Allied Health Care, Monash University, Frankston, Victoria 3199 Australia; 2 Physiotherapy Department, Security Forces Hospital, Riyadh, 11481 Saudi Arabia; 3 School of Primary and Allied Health Care, Faculty of Medicine, Nursing, and Health Sciences, Monash University, Frankston, Victoria 3199 Australia; 4 Discipline of Podiatry, School of Allied Health, Human Services and Sport, College of Science, Health and Engineering, La Trobe University, Melbourne, Victoria 3086 Australia; 5 La Trobe Sport and Exercise Medicine Research Centre, School of Allied Health, Human Services and Sport, College of Science, Health and Engineering, La Trobe University, Melbourne, Victoria 3086 Australia

**Keywords:** Achilles tendinopathy, Function, Capacity, Neuromuscular, Torque, Power, Work

## Abstract

**Background:**

Understanding plantarflexor muscle impairments among individuals with Achilles tendinopathy (AT) may help to guide future research and inform clinical management of AT. Therefore, the aim of this review is to evaluate plantarflexor muscle impairments among individuals with AT and whether plantarflexor muscle function changes following resistance training interventions.

**Methods:**

We searched relevant databases including Cochrane Central Register of Controlled Trials, Ovid (MEDLINE, EMBASE, AMED) and EBSCO (CINAHL Plus and SPORTDiscus) up to September 2020. Studies investigating plantarflexor muscle function were included if they met the following criteria: (1) any study design enabled comparison of plantarflexor muscle function between individuals with and without AT, or the affected and unaffected side of individuals with unilateral AT, and (2) any studies enabled investigation of change in plantarflexion muscle function over time with use of resistance training intervention. We included studies that recruited adults with either insertional or mid-portion AT of any duration. Study selection, quality assessment and data extraction were undertaken independently by two reviewers. Discrepancies were resolved via discussion, or by consulting a third reviewer where necessary. The Joanna Briggs Institute (JBI) critical appraisal tools specific to each study design were used to assess the methodological quality of included studies. Grading the strength of evidence for each outcome was determined according to the quality and number of studies.

**Results:**

A total of 25 studies (545 participants) met inclusion. Participants’ mean age was 40 ± 7 years old. Six studies were high quality for all domains, while the remaining were susceptible to the risk of bias (e.g. selection criteria, reporting findings). This review identified moderate evidence that individuals with AT have impairment in maximal plantarflexor torque (seven studies including one with a mixed population) on their affected side, compared with the unaffected side. Impairments were modest (9% and 13% [pooled effect divided by mean of the unaffected side scores]) and of uncertain clinical importance. The remaining evidence, primarily among individuals with mid-portion AT, showed conflicting impairments for plantarflexor function (i.e. explosive strength and endurance) between sides. There was limited to very limited evidence for improvement in plantarflexor endurance (7% and 23%) but not power or strength (five studies including one with a mixed population for strength) over time, despite individuals undertaking several weeks of resistance training.

**Conclusions:**

Plantarflexor impairments appear more common between sides than compared with control groups but given limitations in the literature further exploration of these relationships is needed.

**Registration:**

PROSPERO Database; number CRD42019100747.

**Supplementary Information:**

The online version contains supplementary material available at 10.1186/s40798-021-00308-8.

## Key Points

- It is not clear whether plantarflexor muscle impairments exist among people with Achilles tendinopathy (AT), and whether plantarflexor muscle function changes with resistance training interventions.
- Apart from impairments in maximal plantarflexor torque, there were conflicting findings for impairments in plantarflexor function in affected and unaffected side comparisons.
- There was conflicting evidence for impairment in all plantarflexor muscle function between people with AT and healthy controls.
- There was also limited to very limited evidence for improvement in plantarflexor endurance but not the other measures (torque, power) over time after a minimum of 12 weeks of resistance training.

## Background

The Achilles tendon is the largest in the body and has an important function in storing and releasing energy during human locomotion [1]. Achilles tendinopathy (AT) is highly prevalent in both active [2] and less active individuals [3] and is characterised by local tendon pathology and pain that can persist for years. Achilles tendinopathy can present either unilaterally or bilaterally, and at either the mid-portion (2 to 6 cm proximal to insertion) or the insertion into the calcaneus. The aetiology of tendinopathy is multifactorial [4], involving both extrinsic factors such as training errors and poor technique and intrinsic factors like strength. Key pathology features include increased cellularity and altered cell phenotype, accumulation of a ground substance, disruption of the collagen matrix, a larger cross-sectional area (CSA) and a decrease in tendon stiffness (force resistance) [5].

Pre-existing plantarflexor impairment has been found to be a risk factor for developing AT [6]. Cross-sectional studies have identified plantarflexor function impairments, including reduced maximal plantarflexor torque output [7–10], rate of force development [11], endurance [7], and altered muscle activation [12–18] among individuals with AT in comparison to the unaffected side or healthy controls. McAullife et al. recently reviewed this literature and concluded that people with AT have strength impairments (i.e. maximal, reactive and explosive strength) compared with the uninjured or asymptomatic side [19]. This conclusion was based on pooled data where comparisons between the affected and unaffected side and comparisons between healthy controls and people with AT were combined. Given this pooling of data, the review by McAuliffe et al. is not able to determine whether impairments exist on the unaffected side or compared with controls. This has important clinical implications; if impairments exist on both sides, clinicians managing AT should not use the unaffected side as a benchmark when setting plantarflexor functional targets [20].

It is also important to determine whether plantarflexor muscle function (and any plantarflexor muscle impairments that exist) can improve over time. Progressive resistance training targeting plantarflexor muscle function is a key component of recommended management for AT [21, 22], yet the extent to which these training interventions influence plantarflexor function is not clear. If current resistance interventions do not improve and recover strength that may signal a need to review current approaches to regaining function among people with AT. Several studies have assessed plantarflexor muscle function before and after resistance training interventions [23–26] but, to the best of the authors’ knowledge, this literature has not been reviewed.

The primary aim of this review is to perform a systematic review of existing literature to identify, critique and summarise the evidence for plantarflexor muscle impairment among individuals with AT. Specifically, we will focus on studies that explore the affected compared with unaffected side, or AT compared with controls. The secondary aim is to review the studies that assess the change in plantarflexor muscle function over time among individuals undertaking resistance training interventions for AT. This knowledge will help clinicians make informed decisions about potential impairments to consider among this clinical group.

## Methods

### Searches

The PRISMA statement for systematic reviews was used to guide the reporting of this review [27]. The review was registered at the International Prospective Register of Systematic Reviews (PROSPERO Database; number CRD42019100747). We searched relevant databases including the Cochrane Central Register of Controlled Trials, Ovid (MEDLINE, EMBASE, AMED) and EBSCO (CINAHL Plus and SPORTDiscus) from inception up to September 2020. The reference lists of all retrieved journal articles were searched for additional articles and forward searches of studies citing eligible studies from our yield were conducted in Google Scholar. The search strategy with keyword terms and specific subject headings within each database was used. Searches spanned three categories: neuromuscular, tendinopathy, and Achilles. For each Keyword search, the Boolean command “OR” was used and categories were linked with the Boolean command “AND”. The search terms for Ovid MEDLINE are shown in Table 1.

**Table 1:** Search terms in MEDLINE database

Neuromuscular OR force OR strength OR RFD OR rate of force development OR proprioceptive deficits OR Proprioception OR constant force OR force match OR MVC OR MVIC OR maximum voluntary contraction OR torque OR power OR muscle bulk OR atrophy AND Tendinopathy AND Achilles Tendon	

### Study Inclusion and Exclusion Criteria

Any study design that addressed our aims was included. This could include studies comparing muscle function between the affected and unaffected side, studies comparing individuals with AT and healthy controls (i.e. cross-sectional, cohort or randomised trial [any type e.g. parallel, factorial]), and studies that enabled investigation of change in these functions over time (i.e. case series, prospective cohort or randomised trial [any type e.g. parallel, factorial]). For the second aim, we were interested in within group change in the measures of interest rather than comparative between group analyses so we could determine firstly whether these measures actually change over time. There was no restriction on the date of publication. Animal studies, case reports, abstracts, non-peer reviewed studies, unpublished studies, letters, reviews, and opinion studies were excluded. Studies published in languages other than English were also excluded.

For prospective studies that enabled the investigation of change in plantarflexor function over time, any resistance training protocol was accepted. This included isometric, eccentric, concentric or isotonic exercise used to treat AT. The resistance training protocol had to be applied for four weeks or longer so that the effects could be observed [28]. For studies that included co-interventions alongside the exercise, such as manual therapy or electrotherapy, we included the exercise only arm if available. Otherwise, we included the study and we planned subgroup analyses to compare findings in studies that did and did not include co-interventions.

#### Types of Participants

We included studies that recruited participants aged eighteen years and older with either insertional or mid-portion AT of any duration. Studies were included regardless of how they diagnosed AT, whether through clinical or imaging, or whether they described diagnosis at all. We planned subgroup analyses to compare effects from studies that diagnosed AT based on established clinical recommendations [21]. Established diagnosis of AT was based on localised pain at the Achilles tendon insertion (insertional) or two to six centimetres above the calcaneus (mid-portion), pain during or after physical activities that loaded the tendon, or pain that was worse in the morning or upon weight bearing after a period of rest [21]. Studies investigating symptomatic AT (imaging pathology on ultrasound or MRI) were eligible for inclusion. Imaging pathology could include tendon thickening, hypoechoic areas or Doppler signal on ultrasound, and thickening or increased signal on MRI [29].

Studies were excluded if they included participants who:

i) Had a complete Achilles tear or rupture, based on presentation or imaging findings.
ii) Had undergone previous Achilles surgery or injection for their currently affected Achilles tendon problem in the last three months.
iii) Had been diagnosed with a neurological disorder (e.g. multiple sclerosis) or systemic inflammatory condition (e.g. rheumatoid arthritis).

Exceptions were made for studies that presented data separately for our population of interest.

#### Types of Outcome Measures

To be included, studies must have one or more measures of plantarflexor function. We included any measure of plantarflexor muscle function including:

i) Strength (e.g. maximal voluntary isometric contraction [MVIC] or maximal voluntary isotonic contraction—peak torque in newtons metre [Nm], isotonic plantarflexor contraction [Nm]or peak force [N]).

ii) Power (e.g. isotonic toe raises or isotonic plantarflexor contraction [joules per second or watts (W)]).

iii) Explosive strength (i.e. rate of producing force [RFD in Ns]).

iv) Endurance or work done (e.g. heel raise work per repetitions [joules (J)]).

v) Motor aspects of proprioception (e.g. force sensing tasks).

Work is often measured over several repetitions, so we categorised this outcome as an endurance measure. Compound measures of plantarflexor muscle function that involved multiple joints and muscles (e.g. squatting, jumping, hopping) were excluded.

### Study Selection

The search yield was downloaded into Endnote version X8 (Thomson Reuters, Philadelphia, USA) and duplicates were removed. Two authors (FH, PV) independently screened titles and abstracts for potentially eligible literature based on a predetermined checklist of inclusion criteria. The full text of studies that were not excluded at this stage was retrieved and independently assessed by the same two authors to determine eligibility. When there was a disparity between the assessors, a third reviewer (PM) was consulted to determine eligibility.

### Study Quality Assessment

The quality assessment was performed independently by two authors (FH, PV). A third reviewer was available to assess conflicts if they occurred (PM), but was not needed. The Joanna Briggs Institute (JBI) critical appraisal tools specific to each study design were used to assess the methodological quality of included studies [30, 31]. The JBI has a range of critical appraisal tools developed specifically for use in systemic reviews that address both quality and bias. All items in the JBI critical appraisal are rated as “Yes”, “No”, “Unclear”, or “Not applicable”. There is no total scoring for these tools. We considered a study to be high quality if all the criteria within a scale were satisfied.

For our first aim—evaluating potential differences in plantarflexor muscle function among individuals with AT (the affected compared with unaffected side, or AT compared with controls)—the JBI case-control appraisal tool was used. This tool assesses representativeness of the control group, definition of the source population, recruitment, validity and reliability of the methods of assessing the condition, identification and adjustment for confounders, exposure period, and statistical analyses.

For our second aim—evaluating change in plantarflexor muscle function following resistance training interventions—the JBI case series appraisal tool was the most appropriate tool since we were interested in within-group change. The tool assesses the inclusion criteria (clarity), standardised, reliable and valid assessment of the condition, whether cases were consecutive (or randomly allocated if it was a trial), inclusion (case series) or retention (trial) of all participants, reporting of participant demographics and clinical information, clear reporting of outcomes, clear reporting of recruitment source, appropriate statistical analyses.

### Data Extraction Strategy

Two authors (FH, PV) independently extracted data onto a separate standard data extraction form. Any discrepancies in study selection or extraction were resolved via discussion, or by consulting a third author where necessary (PM).

The following data were extracted from each study:

i. Study characteristics (first author name, year of publication, study design).
ii. Participant characteristics (mean age [years], mean height [cm], mean weight [kg], sex [number of men], mean duration of symptoms [number of months], activity level, site of injury [insertion or mid-portion], side of injury (unilateral, bilateral), number of participants, inclusion/exclusion criteria).
iii. Outcome of interest, and how the measurement was done.
iv. Mean and standard deviation (SD) of plantarflexor muscle function measure in each group.
v. Clinical pain and function measured by patients' self-report at baseline (e.g. the Victorian Institute of Sports Assessment self-administered Achilles questionnaire (VISA-A), visual analog scale (VAS), or numeric pain rating scale (NPRS).

The standard error or *p*- value and *t*-value were used to calculate the standard deviation, if missing [32]. Otherwise, we contacted authors for missing data; this occurred twice, over two weeks [23, 33]. Mean and SD were extracted from graphs if data were not reported in the manuscript or the authors did not provide the relevant information.

### Data Synthesis and Presentation

Effect sizes—mean difference (MD) and 95% confidence intervals (CIs)—were calculated for continuous data measuring plantarflexor muscle function. Meta-analysis was performed with Cochrane Collaboration statistical software, Review Manager 5.3 (RevMan 2014). Data were pooled where possible to address the first aim—comparison of AT and healthy controls or of the affected and non-affected side. Effect sizes were pooled (MD or standard mean difference [SMD] if data were on different scales) where two or more studies had similar population characteristics (age, activity level, site of injury [mid-portion or insertional]) and utilised the same outcome. We planned to report the back-translated MD and 95% CI when possible by multiplying the SMD value and each confidence band by the SD of the highest-weighted study. We also planned to report—but not pool—data for the second aim investigating the prospective change in plantarflexor function, given that data for this question were pre to post within a group rather than between-group data. The Consensus on Exercise Reporting Template (CERT) was used to evaluate the reporting of exercise doses in interventional studies.

A random effects model was chosen a priori for all analyses, given clinical and methodological heterogeneity are likely to exist between studies. The level of statistical heterogeneity for pooled data was established using *I*^2^ statistic (i.e. interpreted as not important (< 50%), moderate (50–75%) or high (> 75%)) [34]. Where data could not be pooled, we reported effect estimates and 95% CIs in narrative form.

We plotted the percentage change in pain and/or function outcome against various plantarflexor muscle measures over time. For the pain and/or function outcome we used VISA-A (the only disease-specific outcome), otherwise another composite pain and function outcome (if reported) or overall pain measured with VAS or NRS. If there were multiple measures reported for a strength construct (e.g. torque), we selected the one with the highest percentage change. If there were multiple trial arms, we only extracted data for the arms that involved exercise (or exercise only if co-interventions were added to exercise in other arms).

Levels of reported evidence were determined based on a modified version of the van Tulder guidelines [35]. Levels of evidence were determined as the following:

i) Strong evidence: Consistent findings among multiple studies, including at least three high-quality studies.

ii) Moderate evidence: Consistent findings among multiple trials, including at least three moderate/high-quality studies or two high-quality studies.

iii) Limited evidence: Consistent findings among multiple studies, including multiple low/moderate quality studies, or one high-quality study.

iv) Very limited: Findings from one low/moderate quality study.

v) Conflicting evidence: Conflicting findings among multiple studies.

## Results

**Fig. 1:**
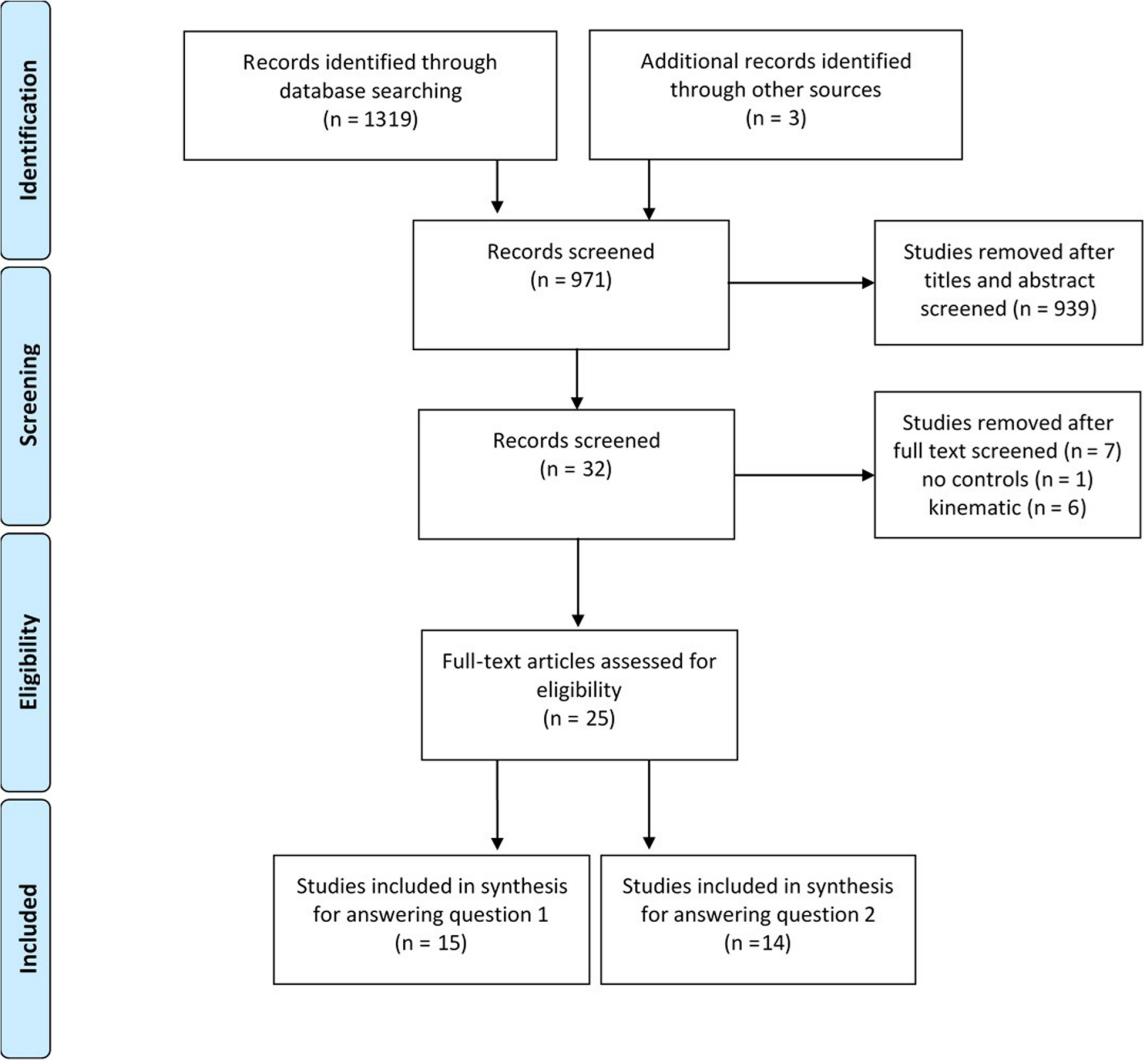
PRISMA flow diagram of the search results

From the 1319 citations in the search yield, a total of 25 studies met our selection criteria. This included 15 studies evaluating the plantarflexor muscle function among individuals with AT (question 1) [8–11, 26, 36–45] and 14 studies evaluating the change in plantarflexor muscle function following resistance training interventions (question 2) [23–26, 33, 39, 41, 45–51]. There were four studies addressing both aims [26, 39, 41, 45]. Figure 1 represents the results of the study selection process.

### Study Characteristics

A total of 545 participants (353 men and 126 women with mean age of 40 years ± 7, mean VISA-A of 60 ± 15 out of 100 points, and BMI of 25 kg/m2 [range 21–30]) were included. One study did not specify the sex distribution of participants [39]. Characteristics of the studies, participants, interventions (prospective studies only) and outcomes are shown in Additional file 4: **Table S1**.

For the first question, all studies adopted a cross-sectional design (i.e. affected vs unaffected (*n* = 11) [11, 26, 36, 37, 39–45], healthy vs AT (*n* = 6)) [8–10, 36, 38, 45]. The majority of the studies included mid-portion AT (*n* = 11). One study included insertional AT [10], two studies included a mixed sample [44, 45] and one did not specify the location of pain [9]. AT diagnosis was based solely on physical examination (i.e. palpation, site of pain, other clinical tests) [8, 9, 39, 45] or physical examination and imaging [10, 11, 26, 36–38, 40–44].

For the second question, studies were mostly randomised trials [24–26, 33, 36, 39, 46–51]. Most studies included only mid-portion AT, though one study did not state the location of AT [24] and one had a mixed population [45]. Resistance training interventions were between 12 weeks and six months in duration and included isotonic loading (i.e. eccentric or concentric-eccentric loading protocols), where load and volume progressed over time (see Table 2 for details).

**Table 2:** Description of exercise interventions using CERT checklist

Study	Alfredson et al. [26, 41]	Boesen et al. [47]	Masood et al. [45]	Mayer et al. [50]	Neeter et al. [51]	Rabusin et al. [46]	Tumilty et al. [48]	Silbernagel et al. [25, 39, 49]	Sancho et al. [23]	Yu et al. [24]	Yu et al. [24]
Intervention	Eccentric exercises	Eccentric exercises	Eccentric exercises	Eccentric exercises	Toe-raises	Eccentric exercises	Eccentric exercises	Toe-raises	Hopping intervention	Eccentric exercises	Concentric exercises
Exercise details	Straight and bend knee heel drop off step	Straight and bend knee heel drop off step	Straight and bend knee heel drop off step	Standardised physiotherapy with eccentric training 10 single 30-min sessions	Two-legged concentric–eccentric toe-raises	Straight and bend knee heel drop off step	Straight and bend knee heel drop off step	Two-legged and one-legged concentric–eccentric toe-raises, and eccentric and fast rebounding toe-raises	4 levels of exercise include isometric, isotonic, jump and hopping	Straight and bend knee heel drop off step	Heel raises using theraband with straightened knee, sit on a chair and lift the heels. Hold onto the wall and lift the heels of both feet and progress to injured one. In addition to Hamstring and calf muscle stretching
Equipment	Backpack and a weight machine	U	Backpack	U	U	Backpack and a weight machine	U	Backpack and a weight machine	U	Bag with dumbbell	Elastic band
Provider	Physical therapist	Physical therapist	U	Physical therapist	Physical therapist	Podiatrist	Physical therapist	Physical therapist	Physical therapist	Research assistant	Research assistant
Delivery	Individually	Individually	Individually	Individually	Individually	Individually	Individually	Individually	Individually	Individually	Individually
Supervision	U	U	U	U	U	U	U	Y	Y	U	U
Reporting of adherence	N	N	N	N	N	Y	N	U	Y	N	N
Motivation strategies	N	N	N	N	N	N	N	N	N	N	N
Decision rules for progressing	Based on pain level	Based on pain level	Based on pain level	U	Based on pain level	Based on pain level	Based on pain level	Based on pain level	Based on load tolerance	Based on pain level	Based on pain level
Illustrations of ex	Y	Y	Y	U	Y	Y	Y	Y	Y	U	U
Home programme content	Y	Y	U	Y	Y	Y	Y	Y	Y	U	U
Nonexercised components	Running activity was allowed if not agg pain	sport activities were allowed if not agg pain	N	U	N	N	U	U	Running activity was allowed if not agg pain	N	N
Incidence of adverse events documented	N	U	N	N	Y	Y	N	Y	Y	N	N
Location of exercises performed	U	U	U	U	U	U	U	U	U	U	U
Dosage*	3, 15 reps, twice a day 7 days/week, for 12 weeks, NS	3, 15 reps, twice a day 7 days/week, for 12 weeks, NS	3, 15 reps, twice a day 7 days/week, for 12 weeks, NS	3, 15, Freq ns, 2-3 time/week, for 4 weeks, NS	2, 30 reps, 3 times/day for 10 weeks, NS	3, 15 reps, twice a day 7 days/week, for 12 weeks, NS	3, 15 reps, twice a day 7 days/week, for 12 weeks, NS	3, 10–15 reps, once a day for 12 weeks to 6 months, NS	Starts with 5 sets of 45 s, three times a day for 12 weeks, BW	3, 15 reps, Freq ns, for 8 weeks, NS	3, 15 reps, Freq ns, for 8 weeks, NS
Tailoring	Generic, intensity was based on the patients’ status	Generic, intensity was based on the patients’ status	Generic, intensity was based on the patients’ status	U	Generic, progression was based on the patients’ status	Generic, intensity was based on the patients’ status	Generic, intensity was based on the patients’ status	Generic, intensity and number of repetitions were based on the patients’ status	Generic, progression was based on the patients’ status	Individual	Individual
Starting level	BW	U	BW	U	U	BW	U	Bilateral	Isometric seated heel raises with BW	Bilateral	Bilateral
Exercise intervention is delivered as planned	U	U	U	U	U	U	U	U	Y	U	U

*Y*,yes; *N*, no; *U*, unclear; *NS*, not specified; *BW*, body weight; *ex.*, exercise; *dosage [sets, repetitions, duration, intensity]

### Plantarflexor Muscle Function Measures

**Fig. 2:**
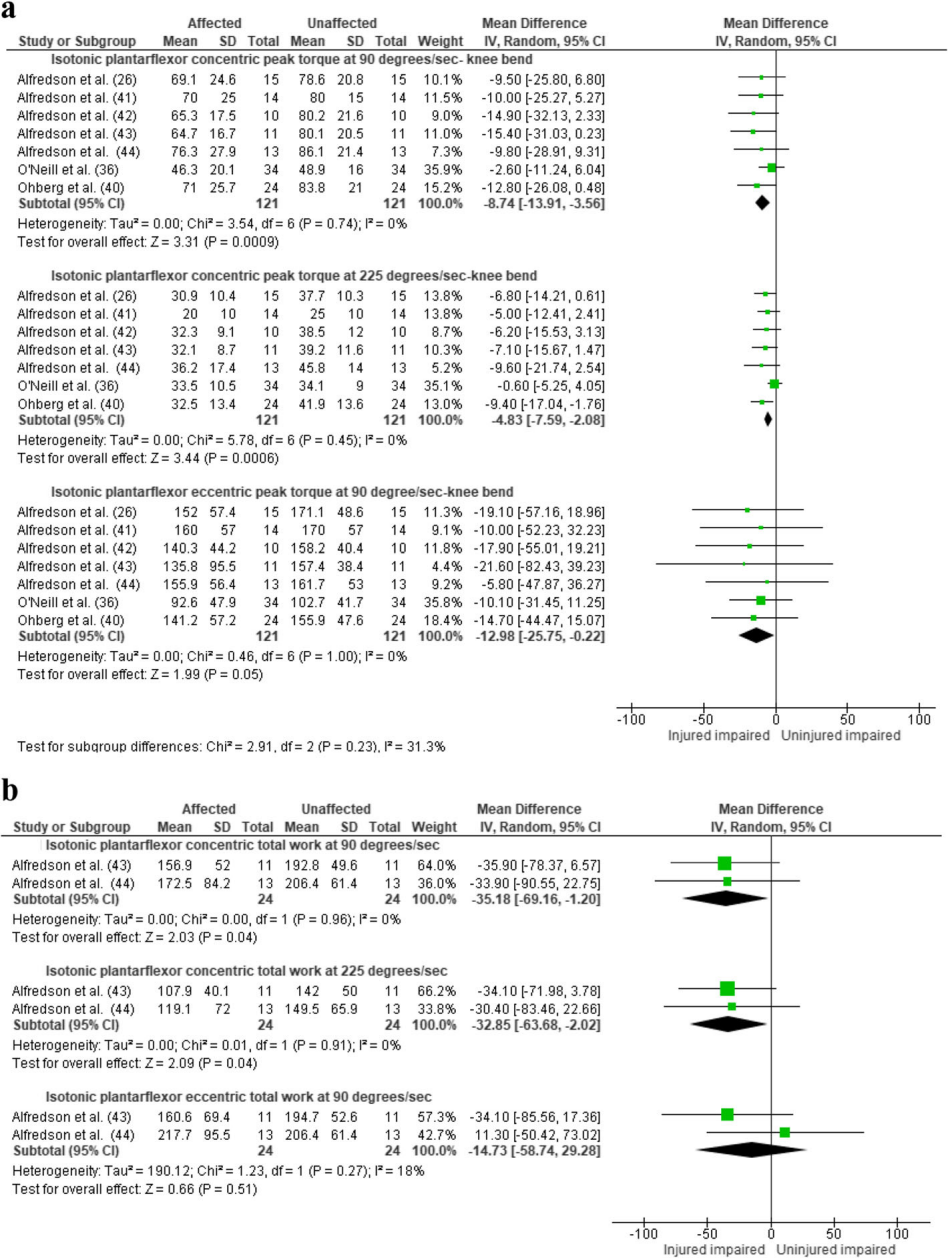
Difference between affected and unaffected sides in **a** isotonic plantarflexion peak torque and **b** endurance. CI, confidence interval; J, Joules

Twelve studies [8, 9, 24, 26, 36, 40–44, 48, 50] assessed plantarflexion peak torque using isokinetic dynamometry and different modes of contraction (i.e. concentric and eccentric), with the knee bent or extended. Four studies [10, 37, 38, 45] assessed maximal voluntary isometric contraction, and one [23] measured six repetition maximum (RM). Two studies [39, 49] assessed plantarflexion power using the isotonic concentric and eccentric heel-raise tests. Two studies [11, 37] measured the plantarflexion explosive strength. Endurance was measured as isotonic total work or average work in ten studies [8, 25, 26, 36, 39, 40, 43, 44, 47, 49] or heel raise test (repetitions to fatigue) in six studies [23, 25, 39, 46, 49, 51]. Three studies did not provide sufficient detail on the outcomes of management [33, 47, 50]. For instance, Mayer et al strength and pain and function data were extracted from graphs because the values were not reported [50]. Data from Horstmann et al. [47] and Boesen et al. [33] were not included in the analysis (Fig. 2) as there were insufficient data reported (in text or in the graphs). No study assessed proprioception.

#### Study Quality Assessment

Quality assessment of the included studies is summarised in Table 3. For the first question, evaluating if there is a difference in plantarflexor function impairment among individuals with AT, four studies scored yes for all items and were considered high quality [11, 36, 37, 40]. Most studies (*n* = 13) appropriately matched the AT and control participants. All studies described the method of measurement of the AT (*n* = 15). One study was at risk of selection bias, as it did not adequately define the criteria for study inclusion and exclusion [9]. One study did not provide details regarding participants with unilateral symptoms; therefore, the validity and reliability of methods used in the assessment of conditions were rated as unclear [39]. Six studies did not clearly report the precise statistical findings (*p* values or 95% CI) or variability data (SD) for the main outcomes [8, 26, 41–44].

**Table 3:** Methodological quality assessment for the included studies

Study	Q1	Q2	Q3	Q4	Q5	Q6	Q7	Q8	Q9	Q10	Total of “yes” scores
Review Question 1: Is there a difference in plantarflexor function impairment among people with AT (the affected compared with unaffected side, or AT compared with controls)?											
Wang et al. [11]	Y	Y	Y	Y	Y	Y	Y	Y	Y	Y	10
Wang et al. [37]	Y	Y	Y	Y	Y	Y	Y	Y	Y	Y	10
Silbernagel et al. [39]	Y	Y	Y	Y	Y	Y	U	U	Y	Y	8
Ohberg et al. [40]	Y	Y	Y	Y	Y	Y	Y	Y	Y	Y	10
Alfredson et al. [41]	Y	Y	Y	Y	Y	Y	Y	Y	Y	U	9
Alfredson et al. [26]	Y	Y	Y	Y	Y	Y	Y	Y	Y	U	9
Alfredson et al. [42]	Y	Y	Y	Y	Y	Y	Y	Y	Y	U	9
Alfredson et al. [43]	Y	Y	Y	Y	Y	Y	Y	Y	Y	U	9
Alfredson et al. [44]	Y	Y	Y	Y	Y	Y	Y	Y	Y	U	9
O'Neill et al. [36]	Y	Y	Y	Y	Y	Y	Y	Y	Y	Y	10
Chimenti et al. [10]	Y	Y	U	Y	Y	Y	Y	Y	Y	Y	9
Masood et al. [45]	Y	Y	U	U	U	Y	Y	Y	Y	Y	7
Child et al. [38]	Y	U	Y	U	Y	Y	Y	Y	Y	Y	8
McCrory et al. [8]	Y	Y	U	Y	Y	Y	Y	Y	Y	U	8
Haglund-Akerlind and Eriksson [9]	N	N	U	U	U	N	N	Y	Y	Y	3
Review Question 2: Is plantarflexor function changed over time following exercise interventions?											
Rabusin et al. [46]	Y	Y	Y	U	U	Y	Y	Y	Y	Y	8
Sancho et al. [23]	Y	Y	Y	Y	Y	Y	Y	Y	Y	Y	10
Boesen et al. [47]	Y	Y	Y	U	Y	Y	Y	Y	Y	Y	9
Masood et al. [45]	Y	Y	Y	U	U	Y	Y	Y	Y	Y	8
Yu et al. [24]	Y	Y	Y	U	Y	Y	Y	Y	Y	Y	9
Horstmann et al. [33]	Y	Y	Y	U	N	Y	Y	N	N	Y	6
Silbernagel et al. [49]	Y	Y	Y	Y	Y	Y	Y	Y	Y	Y	10
Silbernagel et al. [39]	Y	Y	Y	Y	Y	U	Y	Y	Y	Y	9
Tumilty et al. [48]	N	U	U	Y	Y	Y	Y	Y	Y	Y	7
Mayer et al. [50]	Y	Y	Y	U	Y	Y	Y	Y	Y	Y	9
Neeter et al. [51]	Y	Y	Y	U	U	Y	Y	Y	Y	Y	7
Silbernagel et al. [25]	N	Y	N	Y	N	Y	Y	Y	Y	Y	7
Alfredson et al. [41]	Y	Y	Y	Y	Y	Y	Y	Y	Y	U	9
Alfredson et al. [26]	Y	Y	Y	Y	Y	Y	Y	Y	Y	U	9

*Y*, yes; *N*, no; *U*, unclear; *NA*, not applicable

For the second question, evaluating the change in plantarflexor muscle function following resistance training interventions, there were only two high-quality studies out of the included studies [23, 49]. Two studies did not define their inclusion criteria clearly [25, 48]. Seven studies were at risk of selection bias, as they did not indicate a consecutive inclusion in participant recruitment [24, 33, 45–47, 50, 51]. Two studies lacked precision of their statistical analysis, as they did not report the 95% CI of the outcome data [26, 41].

## Quantitative Synthesis for Review Question 1: Is There a Difference in Plantarflexor Function Impairment Among Individuals with AT (the Affected Compared with Unaffected Side, or AT Compared with Controls)?

### Plantarflexor Function in Affected vs Unaffected Side

#### Strength

All findings relate to mid-portion AT, unless stated. There was moderate evidence (seven studies, 121 participants) for lower plantarflexor concentric peak torque at 90°/s (MD = − 8.74 Nm [95%CI = − 13.91 to − 3.56], *I*^2^ = 0%, Fig. 2) and 225°/s (MD = − 4.83 Nm [95%CI = − 7.59 to − 2.08], *I*^2^ = 0%, Fig. 2) and lower plantarflexor eccentric peak torque at 90°/s (MD = − 12.98 Nm [95%CI = − 25.75 to − 0.22], *I*^2^ = 0%, Fig. 2) on the affected side when tested with the knee bent [26, 36, 40–44]. The included studies recruited individuals with mid-portion AT apart from one study that included a mixed population [44].

There was limited evidence (one study, 34 participants) for no difference in plantarflexor concentric peak torque at 90°/s (MD = − 3.50 Nm [95%CI = − 11.23 to 4.23], at 225°/s (MD = − 1.70 Nm [95%CI = − 5.93 to 2.53], and eccentric peak torque at 90°/s (MD = − 0.50 Nm [95%CI = − 17.26 to 16.26], Figure S1) when tested with the knee extended [36].

There was very limited evidence (one study, 11 participants, mixed population of insertional and midportion AT) for no difference in plantarflexor isometric peak force (MD = − 149 N, [95%CI = − 302.92 to 4.92], Figure S1) [45]. Similarly, there was limited evidence (one study, 14 participants) that plantarflexor isometric peak torque was not different between the sides (MD = − 8.20 Nm, [95%CI = − 20.54 to 4.14], Figure S1) [37].

#### Power

There was very limited evidence (one study, 25 participants) for no difference in isotonic concentric toes raise power (MD = − 42 W, [95%CI = − 114.15 to 30.15]), and no difference in and eccentric toes raise power (MD = − 54 W, [95%CI = − 133.06 to 25.06], Figure S1) [39].

#### Explosive Strength

There was limited evidence (one study, 14 participants) for reduction in normalised RFD on the affected side when measured between 0 to 30, 0 to 50 and 0 to 100 ms (MD = − 60 Ns [95%CI = − 99.41 to − 20.59], MD = − 69.6 Ns [95%CI = − 112.92 to − 26.28], MD = − 64.7 Ns [95%CI = − 99.14 to − 30.26], respectively, Figure S1) [37]. There was limited evidence (one study, 17 participants) for no difference in RFD when measured over different time periods (first quarter, half, three quarters and entire time) to reach peak force (MD = − 226.7 Ns [95%CI = − 573.96 to 120.56], MD = − 332.9 Ns [95%CI = − 670.83 to 5.03], MD = − 203.5 Ns [95%CI = − 502.53 to 95.53], MD = − 103.4 Ns [95%CI = − 250.75 to 43.95], respectively, Figure S1) [11].

#### Endurance

There was limited evidence (two studies, 24 participants, one containing a mixed insertional and mid-portion AT population [44]) for a reduction in concentric plantarflexor total work at 90°/s (MD = − 35 .18 Nm [95%CI = − 69.16 to − 1.20]), and at 225 degrees (MD = − 32.85 Nm [95%CI = − 63.68 to − 2.02]) in the affected side, but no reduction in eccentric plantarflexor total work at 90°/s (MD = − 14.73 Nm [95%CI = − 58.74 to 29.28], *I*^2^ = 0%, Fig. 2) [43, 44].

There was also limited evidence (one study, 39 participants) for no difference between sides in plantarflexor total work during maximal concentric-eccentric effort at 90°/s (MD = − 177 Nm [95%CI = − 394.88 to 40.88], Figure S1) [36], and very limited evidence (one study, 24 participants) for no difference in toe raise test for endurance (MD = − 180 J [95%CI = − 747.18 to 387.18], Figure S1) [39]. Similarly, there was very limited evidence (one study, 15 participants) for no difference between sides in concentric plantarflexor average work at 90 degrees (MD = − 5.70 J [95%CI = − 14.31 to 2.91]), at 225°/s (MD = − 3.30 J [95%CI = − 6.96 to 0.36]), and eccentric average work at 90°/s (MD = − 10.80 J [95%CI = − 43.08 to 21.48], Figure S1) [26].

### Plantarflexor Function in AT vs Healthy Controls

#### Strength

There was limited evidence (one study, 39 AT participants and 38 healthy controls) that isotonic plantarflexor concentric peak torque at 90°/s (MD = − 17.30 Nm [95%CI = − 25.73 to − 8.87]), concentric peak torque at 225°/s (MD = − 8.10 Nm [95%CI = − 13.54 to − 2.66]), and eccentric peak torque at 90°/s (MD = -109.70 Nm [95%CI = -129.09 to − 90/31], Figure S2) was lower in those with AT when tested with knee bent [36]. In the same study, with the knee extended, comparable reductions in isotonic plantarflexor concentric peak torque at 90°/s (MD = − 26.10 [95%CI = − 35.45 to − 16.75]), concentric peak torque at 225°/s (MD = − 14.80 Nm [95%CI = − 21.01 to − 8.59]), and eccentric peak torque at 90°/s (MD = -55.50 Nm [95%CI = − 73.46 to − 37.54], Figure S2) were found [36]. Similarly, there was very limited evidence (one study, 10 AT participants and 10 healthy controls, not reported whether insertional or mid-portion AT) that isotonic plantarflexor eccentric peak torque at 30 degree/sec, 60 degree/sec, 120°/s and 180°/s peak torque was lower in AT (MD = − 17.60 Nm [95%CI = − 33.22 to − 1.98], MD = − 21 Nm [95%CI = − 40.04 to − 1.96], MD = − 19.60 Nm [95%CI = − 35.95 to − 3.25], MD = − 19.70 Nm [95%CI = − 32.84 to − 6.56], respectively, Figure S2) [9].

**Fig. 3:**
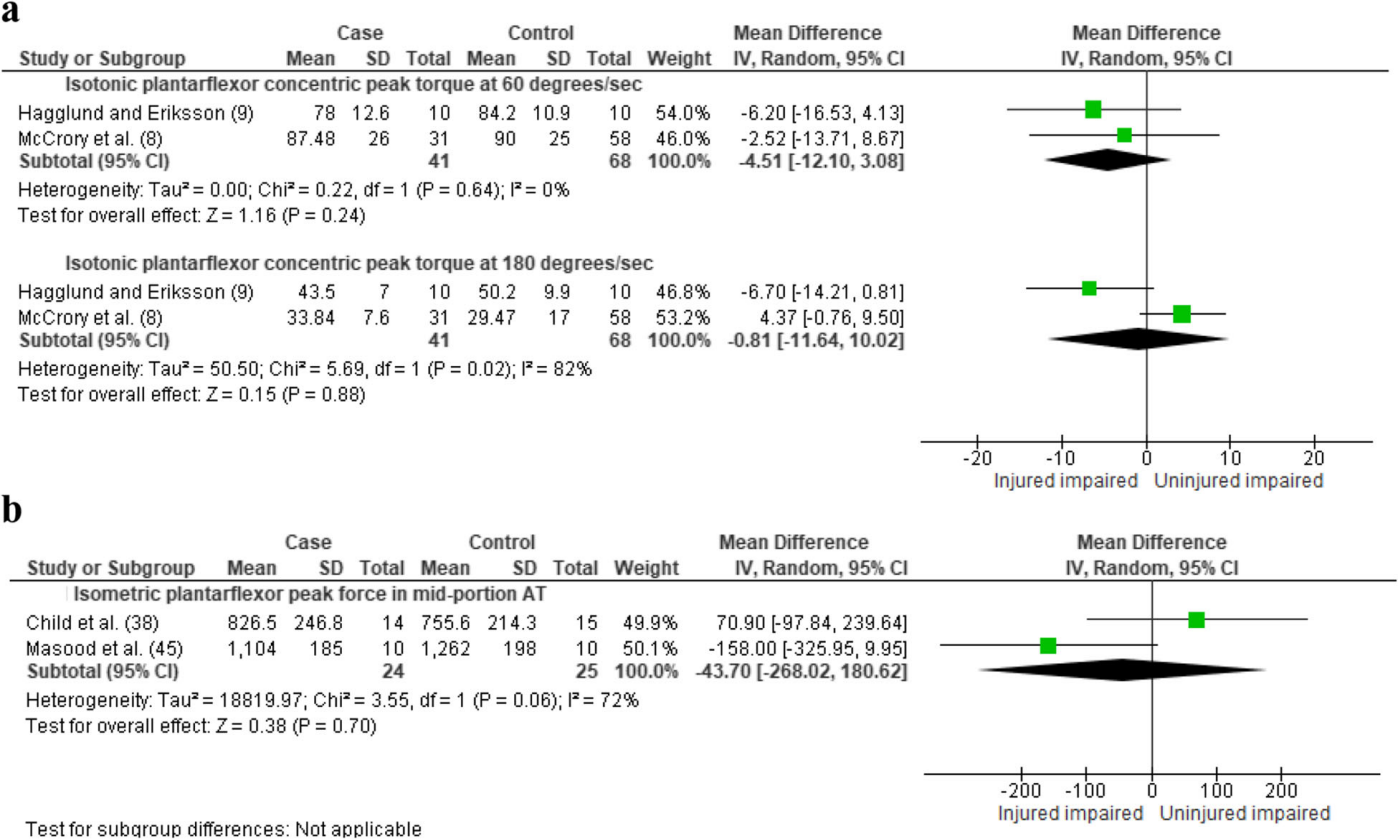
Difference between tendinopathic group and healthy controls in **a** plantarflexor isotonic concentric-eccentric peak torque and **b** plantarflexor isometric peak force. CI, confidence interval

There was very limited evidence (two studies, 41 AT participants and 68 healthy controls) for no difference in plantarflexor peak torque at 60°/s (MD = − 4.51 Nm [95%CI = − 12.10 to 3.08], *I*^2^ = 0%, Fig. 3), and conflicting evidence between the studies at 180°/s (MD = − 0.81 Nm [95%CI = − 11.64 to 10.02], *I*^2^ = 82%, Fig. 3). Substantial heterogeneity may be explained by the uncharacteristically low SD reported in the study by McCrory et al. [8] (confirmed as SD and not standard error in email communication with author).

There was very limited evidence (two studies, 24 AT participants and 25 healthy control) for no reduction in maximal isometric plantarflexion peak force (MD = − 44 N [95%CI = − 268 to 181], *I*^2^ = 72%, Fig. 3) [38, 45]. In contrast, there was very limited evidence that maximal isometric plantarflexion peak torque was lower among individuals with insertional AT (one study, 20 AT participants and 20 healthy controls (MD = − 18.30 Nm, [95%CI = − 34.33 to − 2.27], Figure S2) [10].

#### Power

There was very limited evidence (one study, 31 AT participants and 58 healthy controls) for no difference in isotonic plantarflexion average power (MD = − 8.76 [95%CI = − 19.61 to 2.09], Figure S2) [8].

#### Explosive Strength

No study investigated differences in explosive strength.

#### Endurance

There was limited evidence (one study, 39 AT participants and 38 healthy controls) for a reduction in plantarflexor total work of 20 maximal effort concentric-eccentric plantarflexor at 90°/s (MD = − 613.50 Nm [95%CI = − 833.17 to − 393.83], Figure S2) [36]. Furthermore, there was very limited evidence (one study, 31 AT participants and 58 healthy controls) for no difference in plantarflexor total and average work of 30 repetitions at 180°/s (MD = − 89.10 Nm [95%CI = − 181.70 to 3.50], Figure S2) [8].

## Quantitative Synthesis for Review Question 2: Does Plantarflexion Function Change over Time in Individuals Undertaking Resistance Training Interventions for AT?

### Strength

**Fig. 4:**
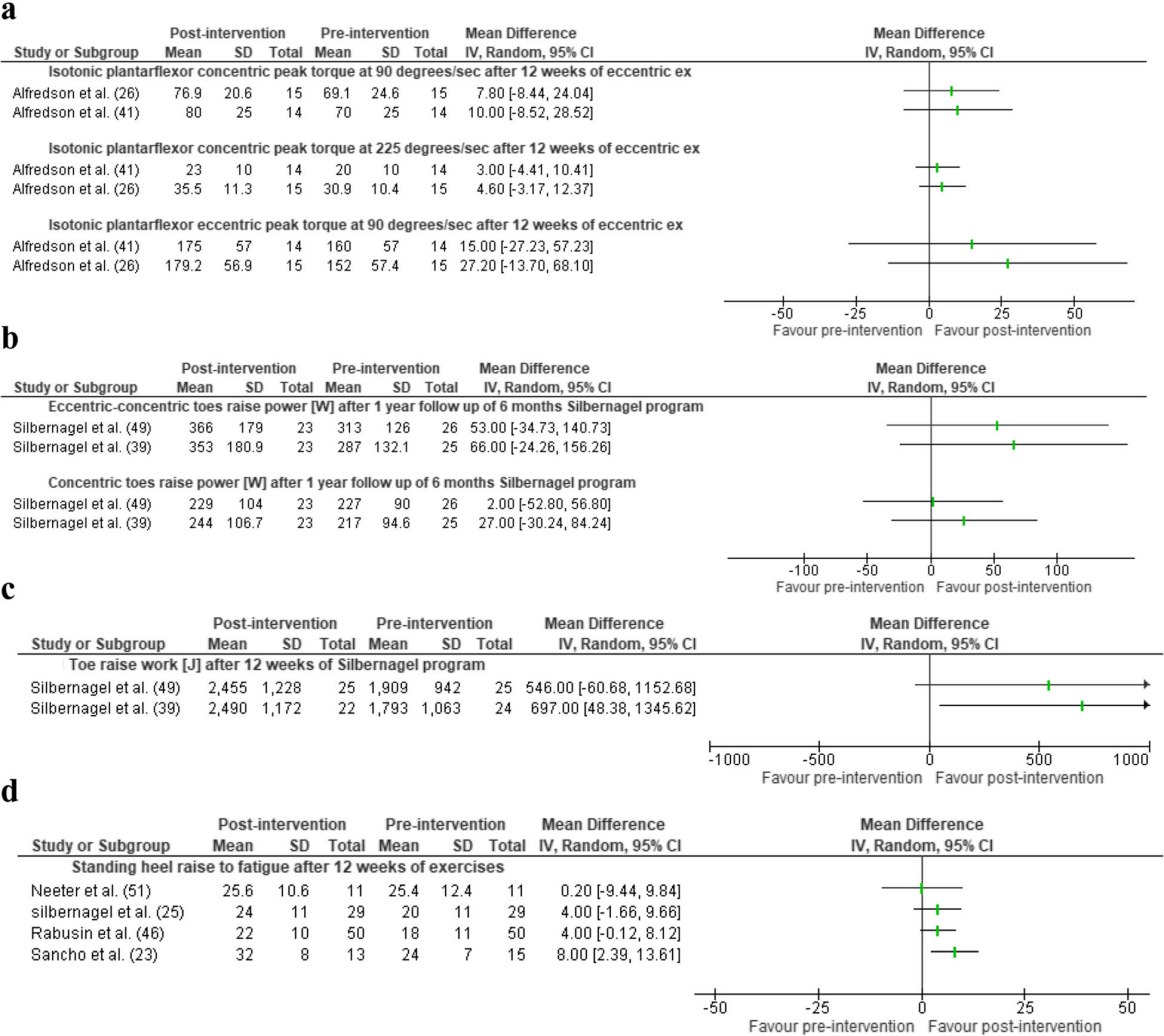
Change over time among individuals with Achilles tendinopathy in **a** isotonic plantarflexor concentric-eccentric peak torque, **b** plantarflexion power, **c** isotonic total work, and **d** heel raise to fatigue. CI, confidence interval; J, Joules

Overall, there was conflicting evidence for improvement in strength. There was limited evidence (two studies, 29 AT participants) for no change in plantarflexor concentric and eccentric peak torque at 90°/s (MD = 8.76 Nm [95%CI = − 3.45 to 20.97] and MD = 21.29 Nm [95%CI = − 8.08 to 50.67, respectively] and concentric peak torque at 225°/s (MD = 3.76 Nm [95%CI = − 1.60 to 9.12], Fig. 4) after 12 weeks of the Alfredson protocol [26, 41]. In contrast, there was limited quality evidence (one study, 15 AT participants) for increase in six RM after 12 weeks of pain guided progressive exercise among runners (MD = 18.70 kg [95%CI = 7 to 30], Figure S3) [23]. Similarly, there was very limited evidence (one study, 20 AT participants) for improved concentric plantarflexion peak torque after 12 weeks of eccentric exercise [48] (MD = 59.20 to 84.20, Figure S3) and very limited evidence (one study, 32 AT participants, not reported whether insertional or mid-portion AT) for improved concentric plantarflexion peak torque at 30°/s after eight weeks of eccentric (MD = 8.7 Nm [95%CI =4.6 to 12.8]) and concentric exercise [24] (MD = 7.97 Nm [95%CI = 2.0 to 13.9], Figure S3). There was very limited evidence (one study, 10 AT participants and 10 healthy controls, mixed population of insertional and mid-portion AT) for no change in maximal isometric plantarflexion peak force among individuals with mid-portion and insertional AT (MD = 194 N [95%CI = − 0.26 to 388], Figure S3) [45].

#### Power

There was limited evidence (two studies, 51 AT participants) for no change in concentric-eccentric plantarflexor isotonic power six weeks and one year after the Silbernagel resistance training programme (MD=26.00 W [95%CI = − 0.29 to 0.80] and MD = 59.32 W [95%CI = − 3.60 to 122.23], *I*^2^ = 0%, respectively, Fig. 4) [39, 49].

#### Explosive Strength

No study investigated the change in explosive strength over time.

#### Endurance

There was limited evidence (two studies, 49 AT participants) for improvement in plantarflexor heel raise test work after 12 weeks of the Silbernagel programme for loading intervention (MD = 616.46 J [95%CI = 173.39 to 1059.54], Fig. 4) [39, 49]. Similarly, there was limited evidence (four studies, 105 mid-portional AT participants) that plantarflexion heel raise test repetitions increased significantly after 12 weeks of resistance training (Silbernagel programme [25, 51] a resistance training and hopping intervention [23] and Alfredson programme [46] (Fig. 4). There was very limited evidence (one study, 32 AT participants, not reported whether insertional or mid-portion AT) that concentric-eccentric plantarflexion endurance (mean torque over 20 repetitions) significantly improved after eight weeks of both concentric and eccentric exercise intervention (MD ranged from 7.68 and13.56 Nm respectively, Figure S3) [24]. In contrast, very limited evidence (one study, 15 AT participants) demonstrated no change in isotonic plantarflexion endurance at 90 and 225°/s with the Alfredson programme at 12 weeks (MD ranged from 2.20 and 4.70 J respectively, Figure S3) [26].

**Fig. 5:**
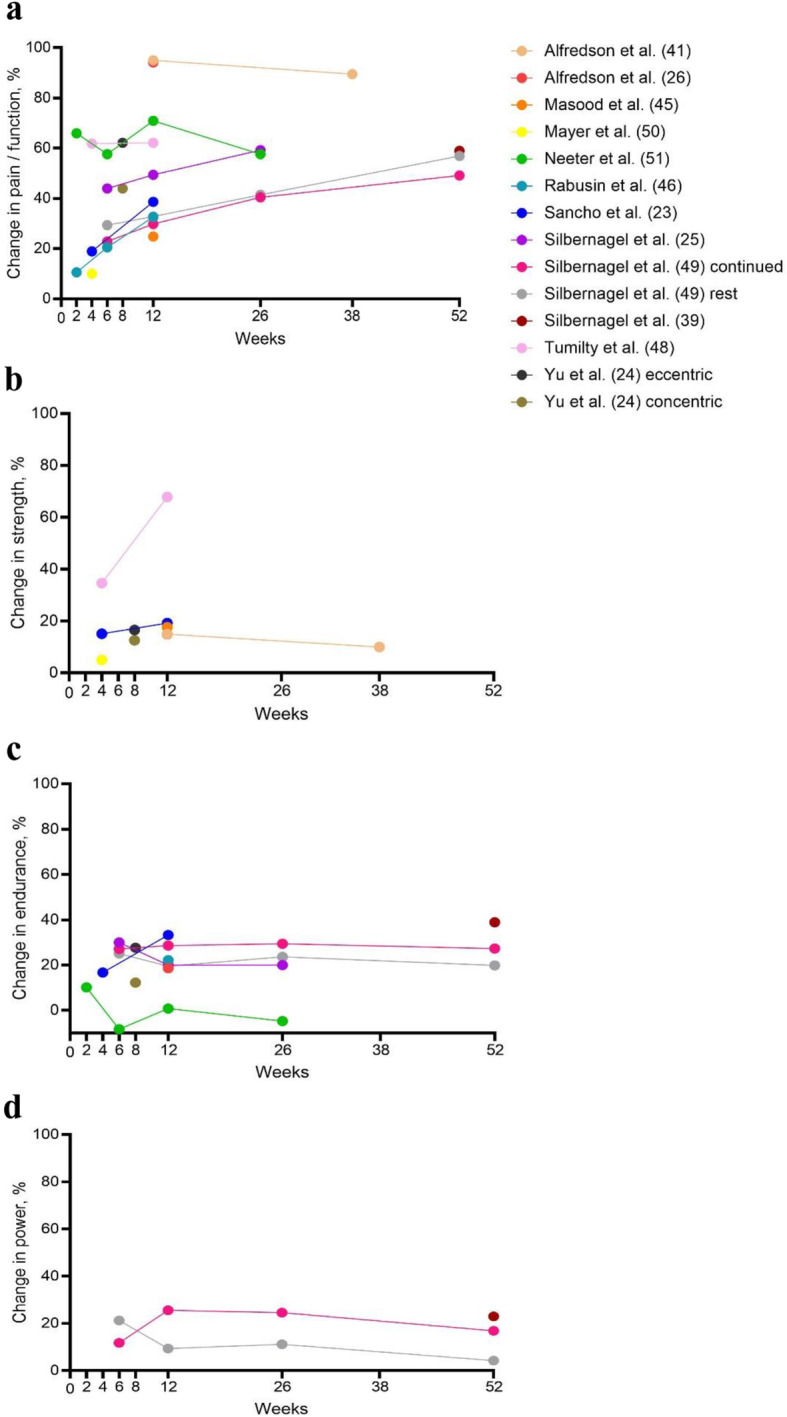
Change (%) in pain/function and various plantarflexor muscle function measures with resistance exercise over time in individuals with Achilles tendinopathy: **a** pain and function measured by the Victorian Institute of Sports Assessment (VISA) self-administered Achilles questionnaire [23, 39, 46, 48, 49], Pain Disability Index [50], visual analog scale (VAS) [24–26, 41] or proportion that had pain with activity [51]; **b** strength, i.e. peak concentric torque [24, 26, 41, 48, 50], isometric torque [45] or repetition maximum [23]; **c** endurance, i.e. work done during isotonic contractions [26, 39, 49], heel raise repetitions [23, 46, 51] or mean concentric torque over repeated repetitions; **d** power, i.e. isotonic heel raise power

Figure 5 shows that the percentage improvement in pain and/or function outcomes ranges from 20.5% to 95.0% at 12 weeks, and further gains appear to be more gradual beyond 12 weeks. In contrast, percentage improvement in plantarflexor muscle function is 14.9% to 19.2% for strength, 0.8% to 33.3% for endurance, and 9.4% to 25.6% for power at 12 weeks.

## Discussion

This review identified moderate evidence that individuals with AT have impairment in maximal plantarflexor torque (seven studies including one with a mixed population) on their affected side, compared with the unaffected side. Impairments were modest (9% and 13% [pooled effect divided by mean of the unaffected side scores]) and of uncertain clinical importance. The remaining evidence, primarily among individuals with mid-portion AT, showed conflicting impairments for plantarflexor function (i.e. explosive strength and endurance) between sides. One study among individuals with insertional AT reported reduced isometric plantarflexor torque (27%) compared with healthy controls. There were no differences for all remaining plantarflexor muscle measures (i.e. isotonic torque, power and endurance) between primarily mid-portion AT and healthy control groups. These findings are different from the conclusion in the previous review by McAullife et al [19], where all plantarflexor functional data—side to side versus healthy to AT comparisons, as well as isometric and isotonic—were combined. The authors of this review reported that there were deficits in plantarflexor function in individuals with AT. Our review provides a more nuanced investigation of how strength may be impaired in AT and suggests that, besides torque and isometric strength impairment on the affected side, other impairments are conflicting and uncertain. There was limited to very limited evidence for improvement in plantarflexor endurance (7% and 23% [pooled effect divided by mean of the unaffected side scores]) but not power or strength (five studies including one with a mixed population for strength) over time, despite individuals undertaking several weeks of resistance training.

Reduction in strength (isotonic and isometric) on the affected side among individuals with AT may be related to apprehension or fear [39, 52]. In a prior trial among individuals with AT, recovery in calf strength was impaired among individuals with greater fear avoidance beliefs [39]. This suggests that fear of loading the affected Achilles tendon may drive some individuals to adopt avoidance-type behaviours, which may manifest during—and influence—maximal strength testing. Impaired strength between sides could also be related to atrophy or reduced muscle activation, which may occur secondary to pain [53]. Lastly, it may be possible that strength impairments were present prior to developing pain [6]. Mahieu et al. investigated prospective risk factors for the development of AT among a cohort of 69 military recruits over six weeks and found a reduction in plantarflexor peak torque for both the right and left leg (MD = − 16.82 Nm [95%CI = − 26.65 to − 6.99] and (MD = − 18.56 Nm [95%CI = − 32.15 to − 4.97], respectively [6]. Currently, there is an incomplete understanding of the mechanisms underlying impaired plantarflexor muscle function among individuals with AT.

The findings of our review are contrary to the theory that individuals with tendinopathy have bilateral strength impairments. Heales et al [54] synthesised the evidence for bilateral sensory and motor deficits involvement in all unilateral tendinopathy, not specifically AT. The meta-analysis of the data demonstrated contralateral sensory (i.e. pressure and thermal pain thresholds, reaction time) and motor (i.e. speed in movement) system deficits in individuals with unilateral pathology. They proposed that there may be motor cortex inhibition affecting strength on both sides and suggested that the unaffected side is not a healthy comparator that can be used in clinical assessment. The difference in findings in our review may be explained by the populations investigated. In the Heales et al. [54] review, 18 out of the 20 studies included were among individuals with upper limb tendinopathy (lateral elbow or rotator cuff tendinopathy). The role of muscle inhibition in the upper limb and lower limb requires consideration in longitudinal studies. It may be that bilateral inhibition and strength impairments manifest for some individuals at varying stages of tendinopathy disease, or that these impairments are specific to the site of tendinopathy.

There were conflicting findings for impairments in plantarflexor muscle function in affected versus unaffected side comparisons, and for the healthy versus controls comparisons. This is surprising, as these measures relate to functional tasks that are impaired among individuals with AT during such as walking [55, 56], running [16, 18, 57] and hopping [12, 49]. For example, calf function relates to walking speed, which has been shown to be impaired in AT [58]. The confidence of our findings may be limited by the limited number of studies, quality of the literature, population size or characteristics. There is also the possibility that differences in the study populations explain the findings. Although studies in this review involved active participants, mostly runners, only two of the six cross-sectional studies specified the duration of AT symptoms. Conflicting findings could also be explained by variability in strength among individuals with AT that may relate to the severity or duration of disease, or other factors [59]. Lastly, conflicting findings could also be because of differences in testing protocols for plantarflexor outcomes. For instance, one study used a heel raise endurance test until fatigue [39], while another study used twenty maximal effort concentric-eccentric plantarflexion contractions [36]. A third used the middle 30 repetitions of 32 repetitions performed at an angular velocity of 180° as an indicator of muscular endurance [26].

Gains in strength after rehabilitation were conflicting. Only endurance measures increased which may be explained by the relatively high-volume resistance programmes prescribed among the studies in this review (15 to 30 repetitions). At face value, it is surprising that we did not observe consistent gains in strength (e.g. torque) because the resistance training prescribed should lead to strength and hypertrophy gains [28]. This finding is less surprising when considered in the context of conflicting plantarflexor function impairments across studies in our review and may reflect the participants having adequate strength when they commenced rehabilitation. Alternatively, the lack of plantarflexor gains in some prospective studies may be related to inadequate prescribed exercise dose (e.g. volume or intensity) or inadequate exercise adherence; both factors were incompletely reported in the included studies (Table 2). Only one out of 14 interventional studies transparently revealed the adherence and fidelity of the resistance training programme [23]. This finding highlights the need to develop strategies for more effective loading programmes to address impairments.

The findings of this review also raise questions about the mechanisms of pain improvement in AT. Despite improvements in pain and function with loading interventions in the included trials, parallel plantarflexor function often improved more modestly (see Fig. 5) and often plantarflexor function improvements were not significant. These findings demonstrate that even with substantial pain and function improvement, improvement in plantarflexor muscle function may be minimal. This questions how important plantarflexor muscle improvements are in improving AT symptoms [60]. It is important to note that we could only observe parallel changes in pain and plantarflexor function at the group rather than individual level [61]. Even if it is not a mechanism for improved pain, recovering plantarflexor muscle function is presumably important to allow individuals to return to activities of daily living such as walking and sport.

An important finding in this review is that plantarflexor impairment is likely to be a heterogeneous finding among individuals with AT, but simply, not everyone with AT will have strength impairments. The challenge that this presents is identifying individuals with impaired strength. Currently, there is an incomplete understanding of what constitutes a plantarflexor muscle impairment for specific populations. Adequate strength is likely to be very individual and depends on activity levels (e.g. athletes of different levels versus non-athletic people), age, and other factors [62]. Efforts to define normal plantarflexor function among the community that includes both healthy individuals and those with lower limb pathology are needed.

### Limitations

There are several limitations related to the literature—particularly, the low individual study quality—which limited robust conclusions. It is important to note that there were differences in diagnostic criteria between studies that may have contributed to heterogeneity in the condition. For example, there may have been a range of pathologies including paratenon and plantaris tendon involvement, or even partial tears, and these pathologies may impact plantarflexor muscle function differently. Further, we identified few studies investigating strength among people with insertional AT which means we have very little certainty about plantarflexor impairments in this population. There were also limitations related to the review. The main review limitation was a limited number of studies that both met our criteria and had similar populations, interventions, and outcomes. This precluded meta-analysis of the data. Another limitation is that we could not identify studies for some of our outcomes of interest. Specifically, although muscle force steadiness has been investigated in the healthy, young and elderly populations, we could not find a study in the AT population. It is also important to note that a majority of studies for the affected vs unaffected side comparison for plantarflexor torque were by the same author’s group (Alfredson et al. [26, 41–44]). Finally, although addressing our aim to investigate within-group change, including non-randomised studies means that biases such as natural history and regression to the mean may threaten the internal validity of any within-group change that was observed.

## Conclusion

There was moderate evidence that individuals with AT have impairments in maximal plantarflexor torque and limited evidence for impairment in concentric endurance on their affected side. However, there was conflicting evidence for other plantarflexor function—explosive strength, power, and other endurance measures—between sides, and for all measures when compared with healthy controls. There was limited to very limited evidence for improvement in plantarflexor endurance but not in strength or power after undertaking 12 weeks of resistance training. There is a need for high-quality studies to investigate plantarflexor impairment in individuals with AT, and to identify optimal interventions to address impairments.

## Supplementary Information

**Additional file 1: Figure S1.** Difference between affected and unaffected sides in a) isotonic plantarflexion peak torque; b) maximal isometric plantarflexion strength; c) isotonic power; d) explosive strength; e) endurance. Abbreviation: CI, confidence interval; RFD, normalised rated of force development; ms, millisecond.

**Additional file 2: Figure S2.** Difference between tendinopathic group and healthy controls in a) isotonic concentric-eccentric plantarflexor peak torque between mid-portion pathological group and healthy controls; b) isometric plantarflexor peak torque between insertional pathological group and healthy controls; c) isotonic power; d) endurance. Abbreviation: CI, confidence interval; J, Joules.

**Additional file 3: Figure S3.** Change over time among individuals with Achilles tendinopathy in a) isotonic plantarflexor concentric-eccentric peak torque; b) isotonic power; c) endurance. Abbreviation: CI, confidence interval; W, Watt unit; J, Joules.

**Additional file 4: Table S1.** Summary of the characteristics of the included studies.

## Data Availability

The datasets generated and/or analysed during the current study are available from the corresponding author on reasonable request.
