# Supplementary figures and images for: Are Plantarflexor Muscle Impairments Present Among Individuals with Achilles Tendinopathy and Do They Change with Exercise? A Systematic Review with Meta-analysis
Source: Sports Med Open. 2021 Mar 10;7:18. doi: 10.1186/s40798-021-00308-8 (PMC7947084; doi:10.1186/s40798-021-00308-8)

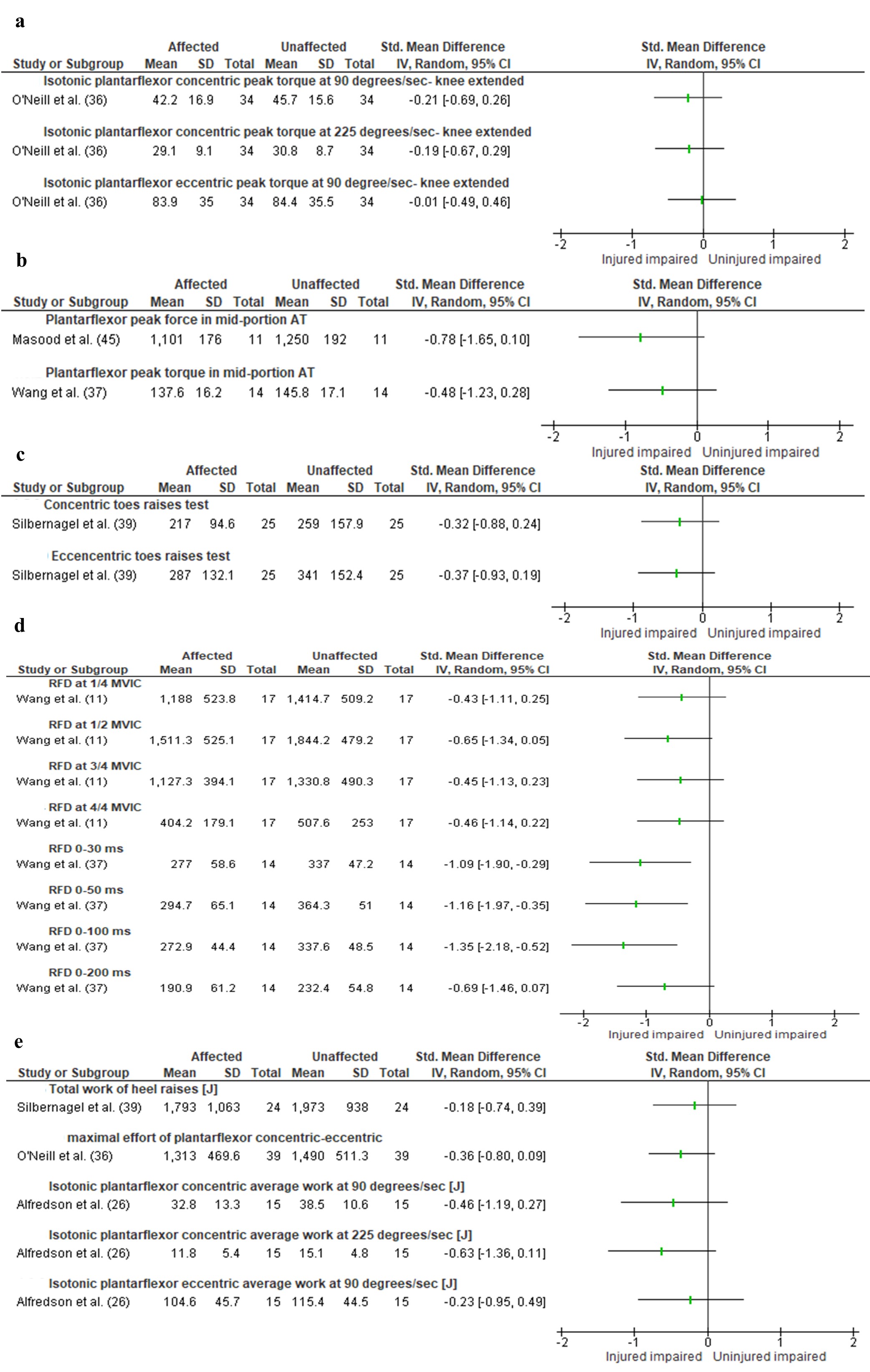

Supplement: Supplementary file 1 — Additional file 1: Figure S1. Difference between affected and unaffected sides in a) isotonic plantarflexion peak torque; b) maximal isometric plantarflexion strength; c) isotonic power; d) explosive strength; e) endurance. Abbreviation: CI, confidence interval; RFD, normalised rated of force development; ms, millisecond. [file 40798_2021_308_MOESM1_ESM.jpg]

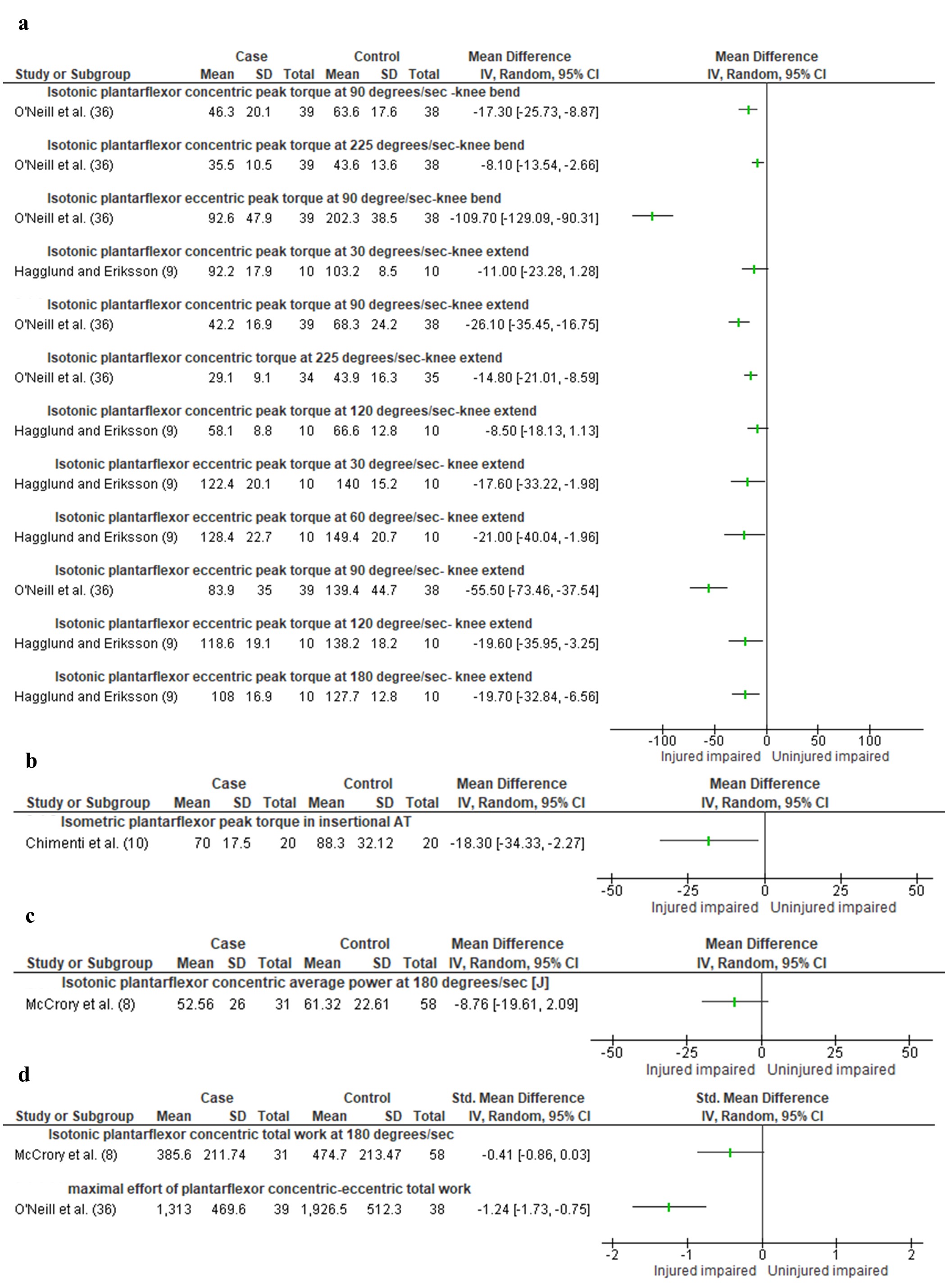

Supplement: Supplementary file 2 — Additional file 2: Figure S2. Difference between tendinopathic group and healthy controls in a) isotonic concentric-eccentric plantarflexor peak torque between mid-portion pathological group and healthy controls; b) isometric plantarflexor peak torque between insertional pathological group and healthy controls; c) isotonic power; d) endurance. Abbreviation: CI, confidence interval; J, Joules. [file 40798_2021_308_MOESM2_ESM.jpg]

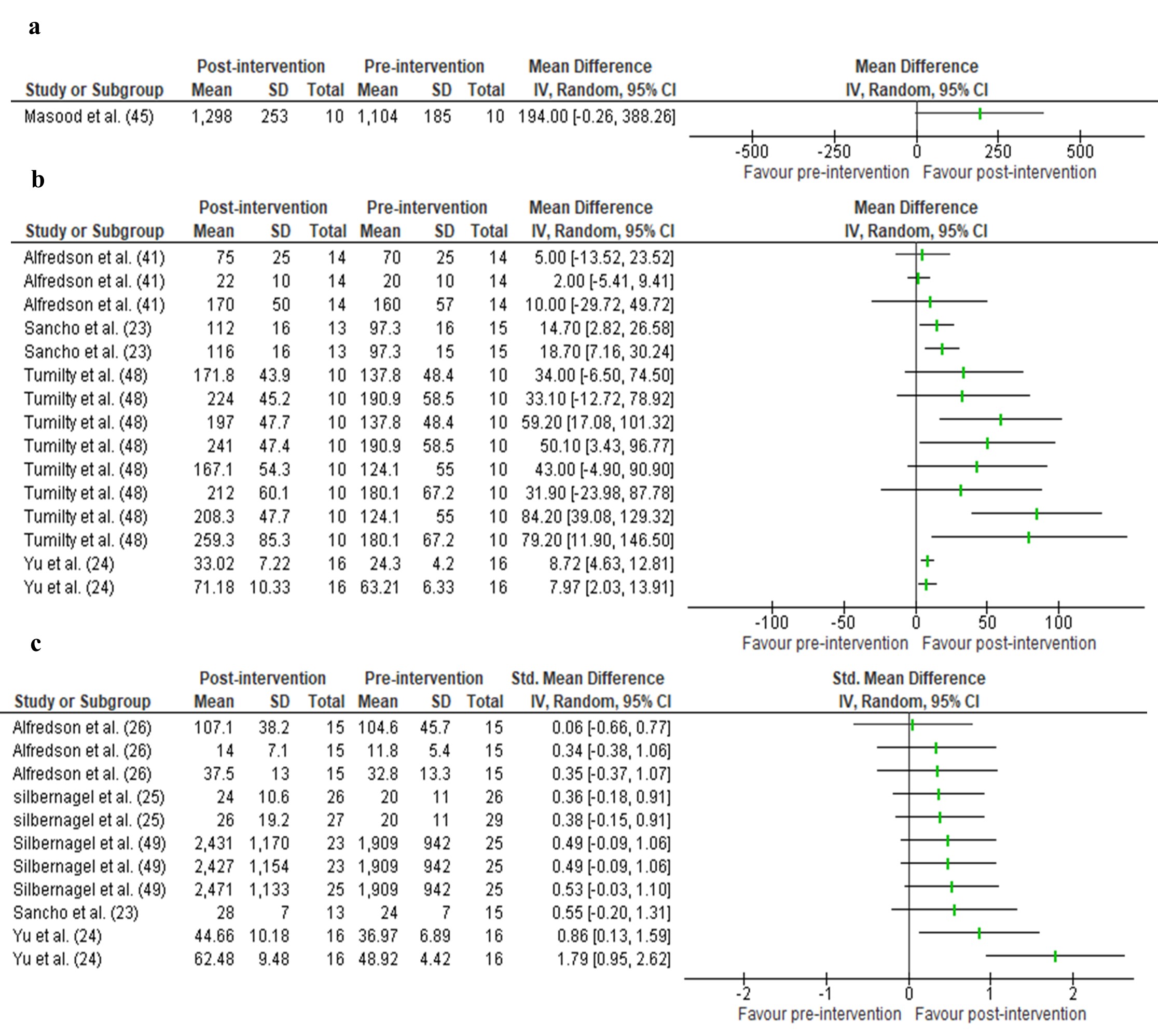

Supplement: Supplementary file 3 — Additional file 3: Figure S3. Change over time among individuals with Achilles tendinopathy in a) isotonic plantarflexor concentric-eccentric peak torque; b) isotonic power; c) endurance. Abbreviation: CI, confidence interval; W, Watt unit; J, Joules. [file 40798_2021_308_MOESM3_ESM.jpg]
