# Supplementary material for: Are Plantarflexor Muscle Impairments Present Among Individuals with Achilles Tendinopathy and Do They Change with Exercise? A Systematic Review with Meta-analysis
Source: Sports Med Open. 2021 Mar 10;7:18. doi: 10.1186/s40798-021-00308-8 (PMC7947084; doi:10.1186/s40798-021-00308-8)
Supplement: Supplementary file 4 — Additional file 4: Table S1. Summary of the characteristics of the included studies. [file 40798_2021_308_MOESM4_ESM.docx]

**Table S1** Summary of the characteristics of the included studies.

| **Author, year** | **Study design** | **Sample size (number of men)/ mean (±SD or range) age [yrs] / height [cm]/ mass [kg]/ pain/function, duration of symptoms [months]/ activity (sport vs recreational)** | | **Unilateral or bilateral or both, site of injury** | **Outcome of interest** |
| --- | --- | --- | --- | --- | --- |
|  |  | Case/ affected | Control/ unaffected |  |  |
| **Review** **Question 1: Is there a difference in plantarflexor function impairment among people with AT (the affected compared with unaffected side, or AT compared with controls)?** | | | | | |
| Wang et al., 2012 (11) | Cross-sectional | 17 (17 men)/ 27.3 ± 2/  183.2 ± 7.1/  75.9 ± 10.8/  VISA-A 70.7 ± 7.8/  5.9 ± 1.3/  13.1 ± 4.2/ athletes [ sprinting, hurdles, badminton, tennis, running, basketball] | Affected vs unaffected side comparison so identical to the affected side | Unilateral, MAT | RFD on the affected and unaffected side. |
| Wang et al., 2011 (37) | Cross-sectional | 14 (10 men)/ 24.2 ± 1.7/  177.3 ± 8.4/  69.2 ± 9/  VISA-A 73.1 ± 4.5/ 5.4 ± 1.6/ athletes [ten  nis, sprinting,  hurdles, heptathlete, long jump, badminton, basketball] | Affected vs unaffected side comparison so identical to the affected side | Unilateral, MAT | Plantarflexor peak torque, RFD, Normalised RFD on the affected and unaffected side.  Normalised RFD was determined as the RFD relative to the maximal torque, expressed as a percent. The onset of voluntary contractions was determined when torque exceeded 7.5 Nm. |
| Silbernagel et al., 2007 (39) | Cross-sectional | 24 (NS)/ 46 ± 8/ NR/ NR/  VISA-A 56 ± 16.5/ 37 ± 67 / NR | Affected vs unaffected side comparison so identical to the affected side | Unilateral, MAT | Concentric and eccentric toes raise test (power in W), and heel raise test for endurance (in J) on the affected and unaffected side. [also, after 12 weeks of exercise program]. |
| O¨ hberg et al., 2001 (40) | Cross-sectional | 24 (17 men)/ 43 (range 26-54)/ 174.1 ± 9.3/ 78 ± 12.2/ Pain NR/ NR/ jogging, running, soccer, tennis, badminton, walking | Affected vs unaffected side comparison so identical to the affected side | Unilateral, IAT (n=NR), MAT (n=NR) | Isokinetic concentric calf-muscle strength (peak torque at 90°/second {5 repetitions}.9 and 225°/second {10 repetitions}), eccentric calf-muscle strength (peak torque at 90°/second {3 repetitions}) on the affected and unaffected side. |
| Alfredson et al., 1999 (41) | Cross-sectional | 14 (12 men)/ 44.2 ± 7.1/ 177.3 ± 8.2/ 77.6 ± 10.0/ VAS 70.5 ± 21.9/ 17.8 (range 3-100)/ jogging | Affected vs unaffected side comparison so identical to the affected side | Unilateral, MAT | Isokinetic concentric calf-muscle strength (peak torque at 90°/second {5 repetitions} and 225°/second {10 repetitions}), eccentric calf-muscle strength (peak torque at 90°/ second {3 repetitions}), on the affected and unaffected side. [also, after 12 and 36 weeks of eccentric exercise program]. |
| Alfredson et al.,  1998 (26) | Cross-sectional | 15 (12 men)/  44.3 ± 7.0/ 176.3 ± 9.4  /77.4 ±10.1/  VAS 81.2 ± 4.8/ 18.3 (range 3-100)/  jogging | Affected vs unaffected side comparison so identical to the affected side | Unilateral, MAT | Isokinetic concentric calf-muscle strength (peak torque and average work at 90°/second {5 repetitions}, and 225°/second {10 repetitions}), eccentric calf-muscle strength (peak torque and average work at 90°/second {3 repetitions}) on the affected and unaffected side. [also, after 12 weeks of eccentric exercise program]. |
| Alfredson et al.,  1998 (42) | Cross-sectional | 10 (5 men)/  40.9 ±10.9/  172.7 ± 9.7/  74.3 ± 11.6/  VAS 78.9 ± 22.1/ 28.6 (range 6-84)/  jogging, soccer, basketball | Affected vs unaffected side comparison so identical to the affected side | Unilateral, MAT | Isokinetic concentric calf-muscle strength (peak torque at 90°/second {5 repetitions} and 225°/second {10 repetitions}), eccentric calf-muscle strength (peak torque at 90°/second {3 repetitions}) on the affected and unaffected side. |
| Alfredson et al.,  1998 (43) | Cross-sectional | 11 (7 men)/ 40.9 ±10.1/ 172.5 ±9.7/  74.4 ±11.2/  VAS 79.7 ±21.6/ 30.9 (range 6-84)/ jogging, soccer, basketball | Affected vs unaffected side comparison so identical to the affected side | Unilateral, MAT | Isokinetic concentric calf-muscle strength (peak torque and total work at 90°/second {5 repetitions} and 225°/second {10 repetitions}), eccentric calf-muscle strength (peak torque and total work at 90°/second {3 repetitions}) on the affected and unaffected side. |
| Alfredson et al.,  1996 (44) | Cross-sectional | 13 (10 men)/ 44.1 ± 8.5/ 175.6 ± 8.8/ 81.5 ± 13.1/ Pain NR/  27.8 ± 20.2/  jogging, soccer | Affected vs unaffected side comparison so identical to the affected side | Unilateral, IAT (n=6), MAT (n=7) | Isokinetic concentric calf-muscle strength (peak torque and total work at 90°/second {5 repetitions} and 225°/second {10 repetitions}), eccentric calf-muscle strength (peak torque at 90°/second {3 repetitions}) on the affected and unaffected side. |
| O'Neill et al.,  2019 (36) | Cross-sectional | 39 (34 men)/ 47 ±11.8/  177 ± 6.8/  77 ± 12.1/  VISA-A 56 ± 17.8/ NR/ normally running >2 times per week | Healthy 38 (35 men), matched by age and sex (+/-4%)/ 44 ± 9.9/ 175 ± 8.1/ 70.4 ± 10.3/ VISA-A 100 ± 0/NA/ >2x/week | Unilateral, MAT | Isokinetic concentric and eccentric muscle torque at 90˚/sec, concentric torque at 225˚/sec, and endurance capacity (20 maximal effort concentric/eccentric contractions) of the plantar flexors on case-control and between sides. |
| Chimenti et al.,  2016 (10) | Cross-sectional | 20 (9 men)/ 58.6 ± 7.8/ 170 ± 10/ 87.5 ± 17.5/ VISA-A 47.6 ± 26.8/ 10 (range 3-15 years)/ NR | 20 (9 men) age and sex matched/ 58.2 ± 8.5/ 170 ± 10/ 80.3 ± 16.0/ VISA-A NR/ NA/ NR | Unilateral, IAT | Plantarflexor torque on case-control. |
| Masood et al.,  2014 (45) | Cross-sectional | 10 (7 men)/ 27.9 ± 4 /175.4 ± 5/  66.5 ± 5/  VISA-A 63.8 ± 19/ 10.4 ± 8 (range 2 -25)/ NR | Healthy 10 (7 men), (± 5%) matched demographics 27.6 ± 4.1/  174.3 ± 3.1/  68.7 ± 6.6/  VISA-A NR/ NA/ NR | Unilateral, IAT (n=NR), MAT (n=NR) | Plantarflexor force on case-control and between sides. [also, after 12 weeks of eccentric exercise program]. |
| Masood et al.,  2014 (15) | Cross-sectional | 11 (7 men)/ 28 ± 4/174 ± 6/ 66 ± 6/  VISA-A 64 ± 18/ 9.8 ± 8 /  average of 4.7 exercise sessions/week/ running, long jump, high jump, ice-hockey | Healthy 10 (7 men), anthropometrically matched/  28 ± 4/ 173 ± 4/ 67 ± 6/  VISA-A NR/ NA/  physically active (NS) for 2.4 times per week | Unilateral, IAT (n=NR), MAT (n=NR) | Plantarflexor force on case-control and between sides.  *Note: confirmed from communication with the authors that it is the same cohort in both Masood et al studies (aside from 1 participant).* |
| Child et al., 2010 (38) | Cross-sectional | 14 (all men)/ 40 ± 8/ 177 ± 6/ 80 ± 9/ VAS NR/ NR/ jogging, running, soccer, tennis, badminton, walking | 15 (all men)/ 35 ±9 / 178 ± 5/ 79 ± 11/  VAS NR/ NA/ jogging, running, soccer, tennis, badminton, walking | Unilateral, MAT | Plantarflexor force on case-control. |
| McCrory et al.,  1999 (8) | Cross-sectional | 31 (sex is NR)/ 38.4 ± 1.8/  174.5 ± 1.35/ 71.43 ± 1.74/ NR/ NR/ runners 52.1 ± 4.68 km/week | Healthy 58 (sex is NR)/ 34.5 ± 1.2/ 174.5 ± 1.04/ 70.03 ± 1.27/ NR/ NA/ runners 44.5 ± 2.85 km/week | Unilateral, MAT | Isokinetic peak plantarflexor torque at 60˚ second and total work, endurance ratio and average power at 180°/second. |
| Haglund Y and Eriksson E, 1993 (9) | Cross-sectional | 10 (all men)/ NR/ NR/ NR/ NR/ NR running | 10 (all men)/ NR/ NR/ NR/ NR/ running | Unilateral,  NR | Isokinetic concentric and eccentric muscle torques of the gastrocnemius-soleus complex were recorded at 30˚, 60˚, 120˚ and 180°/second {3 repetitions} on case-control. |
| **Review Question 2: Is plantarflexor function changed over time following exercise interventions?** | | | | | |
| Rabusin et al., 2020 (46) | RCT | Experiment  Group (heel lifts)  50 (24 men)/ 46.1 ± 9/ 171.9 ± 9.1/ 85.6 ± 17.7/ VISA-A 57 ± 15.4/ 18 (range 6- 36)/ NR | Control  Group (eccentric exercise)  50 (24 men)/ 45.6 ± 9.8/ 172.6 ± 10.7/  89.9 ± 22 / VISA-A 53.3 ± 17.1/ 22.5 (range 8- 36)/ NR | Unilateral, MAT | Concentric and eccentric heel raise to fatigue at baseline and 12 weeks of eccentric exercise intervention. |
| Sancho et al., 2019 (23) | Case series  (Feasibility) | 15 (all men)/ 37.86 ± 8.83/178.50 ± 5.99/ 87.19 ± 10.57/  VISA-A 62.23 ± 17.36/ NR/ NR** | NA | Both, MAT | Standing heel raise to fatigue and seated heel raise 6 repetition maximum at baseline, 4 and 12 weeks. |
| Boesen et al., 2017 (47) | RCT | Experiment  Group (eccentric  strengthening exercise combined with high-volume injection)  20 (all men)/ 41.9 ± 12.2/ 182.6 ± 7.1/ 83.6 ± 19.6/ VISA-A 52.5 ± 12.8/ 24.7 ± 17.1/ NR  Experiment  Group (eccentric  strengthening exercise combined with platelet-rich plasma)  20 (all men)/ 43.1 ± 8.1/ 183.1 ± 19.4/ 86.5 ± 20.6/ VISA-A 58.1 ± 12.4/ 27 ± 34/ NR | Control  Group (eccentric  strengthening exercise combined with placebo [saline under skin])  20 (all men)/ 40.9 ± 6.6/ 183.5 ± 20.4/ 89.7 ± 22.1/ VISA-A 59.2 ± 10.1/ 30.8 ± 37.4/ NR | Unilateral, MAT | Heel raise to fatigue (in J) at baseline, 12 and 24 weeks of eccentric exercise intervention. |
| Yu et al., 2013 (24) | RCT | Experiment  Group (eccentric  strengthening exercise)  16 (all men)/ 20.14 ± 1.84/ 171.08 ± 3.25/ 63.53 ± 4.46/ VAS 5.72 ± 0.89/ 11.28 ± 2.9/ NR | Control  Group (concentric  strengthening exercise)  16 (all men)/ 20.40 ± 1.27/  172.53 ± 2.04/  64.25 ± 6.41/  VAS 5.72 ± 0.79/ 12.12 ± 1.32/ NR | Unilateral, NR | Isokinetic concentric and eccentric muscle torque at 30˚/sec, and endurance capacity (20 maximal effort concentric/eccentric contractions at 120˚/sec) of the plantar flexors at baseline and 8 weeks of exercise intervention. |
| Horstmann et al., 2013 (33) | RCT | Experiment  Group (eccentric  strengthening exercise)  19 (10 men)/ 45.7 ± 8.5/ 173.3 ± 8.9/ 74.5 ± 10.3/ VISA-A 72.9 ± 31.4/ NR/ NR | Control Group (wait and see and vibration) | Both, MAT | Isokinetic concentric at 60˚/sec, and concentric-eccentric muscle torque at 20˚/sec at baseline and after 12 weeks of intervention. |
| Tumilty et al., 2008 (48) | RCT  (Feasibility) | Experiment  Group (eccentric  strengthening exercise with active laser treatment)  10 (3 men)/ 41.4 ± 7.6/  NR/ NR/  VISA-A 57 ± 16.7/ NR/ NR | Control Group: (eccentric  strengthening exercise with placebo laser treatment)  10 (6 men)/ 42.5 ± 8.5/ NR/ NR/  VISA-A 56.3 ± 19.8/ NR/ NR | NR, MAT | Isokinetic concentric and eccentric muscle torque at 90˚/sec, at baseline and after 12 weeks of intervention. |
| Silbernagel et al., 2007 (49) | RCT | Experiment  Group (exercise training)  19 (12 men)/ 44 ± 8.8/ 179 ± 9/ 80.7 ± 15/ VISA-A 26 ± 13/ 48 ± 84.5/ NR | Control  Group (active rest during the first 6 weeks of rehabilitation)  19 (8 men)/ 48 ± 6.8/ 177 ± 8/ 78.7 ± 11.6/  VISA-A 23 ± 16/ 24.4 ± 40.8/  NR | Both, MAT | Concentric and eccentric toes raise test (power in W), and heel raise testfor endurance (in J) at baseline and 6,12,24,48 weeks of progressive Achilles tendon loading strengthening program. |
| Mayer et al., 2007 (50) | RCT | Experiment  Group (eccentric-concentric  strengthening exercise)    31 (all men)/ 41 ± 5.9/ 176 ± 5.9/  70.7 ± 7/  Pain NR/ 17.3 ± 18.7/ running | Control Group (no treatment and those wearing insoles) | Unilateral, MAT | Isokinetic eccentric peak torque at baseline and 4 weeks of intervention. |
| Neeter et al, 2003 (51) | RCT | Experiment  Group (strengthening exercises based on Silbernagel program combined with iontophoresis with dexamethasone)  14 (9 men)/ 38 ± 15.6/ NR/  NR / Pain NR/ jogging, Aerobics, weightlifting, walking, badminton, table tennis, soccer, floorball, and volleyball | Control  Group (strengthening exercises based on Silbernagel program combined with iontophoresis with saline)  11 (6 men)/ 39 ± 3.9/ NR/  NR / Pain NR/ jogging, Aerobics, tennis, walking, soccer, and boxing | Unilateral, MAT | Concentric and eccentric heel raise to fatigue at baseline and 6, 12 weeks, 6 months, and one year of exercise intervention. |
| Silbernagel et al., 2001 (25) | RCT | Experiment  Group (structured exercise program)  22 (17 men)/ 47 ± 14.7/ 178 ± 7.8/ NR/  VAS 30 (median 49)/ 20 ± 25.4/ jogging, aerobics, ice hockey, tennis, weightlifting, golf, walking, badminton, table tennis | Control  Group (common practice exercise program)  18 (14 men)/ 41 ± 10.2/ 178 ± 8.2/  NR/ VAS 27 (median 27)/  41 ± 55.9/  jogging, aerobics, walking, soccer, handball, floorball | Both, MAT | Concentric and eccentric heel raise to fatigue at baseline and 6,12, 24 weeks of exercise intervention. |

NR, not reported ; NS, not specified ; NA, not applicable; MAT, Mid-portion Achilles tendinopathy ; VAS, 10-cm visual analog scale that reported the amount of pain during activity (running); VISA-A, he Victorian Institute of Sport Assessment-Achilles questionnaire; RFD, rated of force development; * most affected side was chosen for data collection by the authors; ** physical activity not reported at baseline, although five participants returned to sport during the program, including football, basketball, rugby, Australian football
